# Do cognitive interventions improve general cognition in dementia? A meta-analysis and meta-regression

**DOI:** 10.1136/bmjopen-2014-005247

**Published:** 2015-04-02

**Authors:** J D Huntley, R L Gould, K Liu, M Smith, R J Howard

**Affiliations:** Department of Old Age Psychiatry, Institute of Psychiatry, Psychology and Neuroscience, King's College London, London, UK

**Keywords:** GERIATRIC MEDICINE

## Abstract

**Objectives:**

To review the efficacy of cognitive interventions on improving general cognition in dementia.

**Method:**

Online literature databases and trial registers, previous systematic reviews and leading journals were searched for relevant randomised controlled trials. A systematic review, random-effects meta-analyses and meta-regression were conducted. Cognitive interventions were categorised as: cognitive stimulation (CS), involving a range of social and cognitive activities to stimulate multiple cognitive domains; cognitive training (CT), involving repeated practice of standardised tasks targeting a specific cognitive function; cognitive rehabilitation (CR), which takes a person-centred approach to target impaired function; or mixed  CT and stimulation (MCTS). Separate analyses were conducted for general cognitive outcome measures and for studies using ‘active’ (designed to control for non-specific therapeutic effects) and non-active (minimal or no intervention) control groups.

**Results:**

33 studies were included. Significant positive effect sizes (Hedges’ g) were found for CS with the mini-mental state examination (MMSE) (g=0.51, 95% CI 0.29 to 0.69; p<0.001) compared to non-active controls and (g=0.35, 95% CI 0.06 to 0.65; p=0.019) compared to active controls. Significant benefit was also seen with the Alzheimer's disease Assessment Scale-Cognition (ADAS-Cog) (g=−0.26, 95% CI −0.445 to −0.08; p=0.005). There was no evidence that CT or MCTS produced significant improvements on general cognition outcomes and not enough CR studies for meta-analysis. The lowest accepted minimum clinically important difference was reached in 11/17 CS studies for the MMSE, but only 2/9 studies for the ADAS-Cog. Additionally, 95% prediction intervals suggested that although statistically significant, CS may not lead to benefits on the ADAS-Cog in all clinical settings.

**Conclusions:**

CS improves scores on MMSE and ADAS-Cog in dementia, but benefits on the ADAS-Cog are generally not clinically significant and difficulties with blinding of patients and use of adequate placebo controls make comparison with the results of dementia drug treatments problematic.

**Strengths and limitations of this study**

- This is a comprehensive meta-analysis of cognitive interventions in Alzheimer's disease (AD), specifically examining efficacy of interventions compared to active and non-active control groups.
- By examining common clinically used general cognitive outcome measures, we question whether cognitive interventions lead to clinically important differences.
- This meta-analysis highlights important limitations in the literature such as difficulties with blinding of patients and use of adequate placebo controls, which make comparison with the results of dementia drug trials problematic.

## Introduction

Cognitive interventions are widely used to aid cognitive function in people suffering from dementia. There are three main approaches, which have been summarised by Clare and Woods.^1^ Cognitive training (CT) involves repeated practice of a standardised task that targets a specific cognitive function. The assumption is that such ‘training’ will lead to an improvement in the cognitive domain trained, and potentially to generalised improvements in cognitive function. Such CT is usually delivered individually, and may be computerised or non-computerised. CT is often adaptive, allowing an increase in task difficulty as expertise develops.

Cognitive stimulation (CS) refers to a more non-specific approach, where a range of different activities are used to engage and stimulate the individual. There may be components of reminiscence therapy, reality orientation, social activity and sensorimotor activities. Emphasis is on the involvement of multiple cognitive domains rather than the targeting of one specific cognitive function. It is normally a group rather than individual intervention, with a significant emphasis on social interaction.

Cognitive rehabilitation (CR) differs in that it takes a particular impaired ability as the starting point and, using a person-centred approach, seeks to find solutions or approaches that enable the individual to perform the desired function or task (table 1).

**Table 1:** Definitions of interventions and control groups (adapted from Clare and Woods^1^)

Cognitive training	Repeated guided practice
	Uses standardised tasks
	Theoretically motivated strategies
	Range of difficulties (adaptive)
	Aim for improvement in isolated cognitive domain with possibility of generalisation to non-trained task
Cognitive stimulation:	Wide range of activities
	Group format
	Significant emphasis on social interaction
	Aim for general improvement in cognitive function
	Not adaptive
	Significant use of reality orientation or reminiscence therapy
Cognitive rehabilitation:	Individualised goals
	Aim to improve everyday function/ADLs
	Compensatory approach
Control group	
Active control group	Intervention matched for time/social interaction Intervention contains cognitive content not directly related to cognitive outcome measure
Non-active control group	Waiting list/treatment as usual or minimal intervention not matched for time/social interaction/no specific cognitive content

ADLs, activities of daily living.

The National Institute for Health and Care Excellence (NICE) guidelines recommend the use of CS,^2^ however, there is a lack of clarity over the effectiveness of these interventions in terms of stabilisation or improvement in cognition. A Cochrane meta-analysis of CS included 15 randomised controlled trials (RCTs) and concluded that CS significantly improved general cognitive outcomes such as the mini-mental state examination (MMSE,^3^ mean difference=1.74, 95% CI 1.13 to 2.36, p<0.001) and Alzheimer's disease Assessment Scale-Cognition (ADAS-Cog,^4^ mean difference=2.27, 95% CI 0.99 to 3.55, p=0.0005).^5^ A Cochrane review of 12 RCTs investigating CT or CR reported no significant improvements on any cognitive outcome measure.^6^ Neither of these meta-analyses examined the effects of including active or non-active control conditions on effect size.

By contrast, Sitzer *et al*^7^ reviewed 5 non-RCTs and 12 RCTs of cognitive interventions in dementia, defined by whether compensatory or restorative approaches were used. Overall effect sizes of (Cohen's) d=0.37 (SD 0.45) for restorative and d=0.40 (SD 0.46) for compensatory interventions on general cognitive outcomes were reported.^7^ Studies that compared intervention to waiting list controls tended to produce greater effect sizes (d=0.53, SD=0.47) than those using attention-controlled placebo controls (d=0.36, SD=0.58), although this difference in effect size was non-significant (p=0.511).

Most recently, Kurz *et al*^8^ found significant standardised mean differences (SMD) on the MMSE (SMD 0.21, 95% CI 0.03 to 0.39, p=0.02) and ADAS-Cog (SMD −0.3, CI −0.48 to −0.13, p=0.0005) for CS, but not for CT and CR in a meta-analysis of RCTs in dementia and mild cognitive impairment (MCI). These authors concluded that there was no convincing evidence that these cognitive score changes generalised to any clinically significant improvements in quality of life or activities of daily living (ADLs).^8^

Consideration of these meta-analyses highlights the limitations of the evidence base. Methodological difficulties, such as a lack of suitable control interventions^9^ and failure to maintain complete blinding to allocation, can mean that factors such as increased attention, socialisation or motivation could contribute to observed changes, or that participant and investigator placebo effects may operate. This would be particularly expected for CS interventions which are often less specific in nature and encompass more social activities.

The current analysis therefore aimed to:

1. evaluate the efficacy of cognitive interventions with consideration of the use of ‘active’ and ‘non-active’ controls. Active controls comprise of interventions designed to control for non-specific therapeutic effects, including time, attention and non-specific input from research or clinical teams (eg, social support, psychoeducation, discussion groups or non-directed activities). Non-active controls consist of treatment as usual (TAU), waiting list conditions, or a minimal intervention not matched for time, social interaction or with no specific cognitive content (table 1);
2. examine effects on commonly-used clinical outcomes of general cognitive function (MMSE and ADAS-Cog) and consider whether these met published criteria for minimum clinically important differences (MCIDs);
3. conduct meta-regression analyses to examine associations between effect sizes and variables that may influence the efficacy of cognitive interventions, such as format, setting or intensity of intervention, severity of dementia or study quality.

## Methods

### Selection of studies

Online literature databases and trial registers (Web of Knowledge, Cochrane Collaborative Central Register of Controlled Trials, and PubMed/Medline) were searched on 6 June 2013 using the terms in table 2. Previous meta-analyses and systematic reviews of cognitive interventions in dementia^1^ ^5–8^ were also searched, in addition to leading journals.

**Table 2:** Search terms

Intervention terms	“cognitive stimulation” OR “cognitive rehabilitation” OR “cognitive training” OR “cognitive therapy” OR “cognitive retraining” OR “cognitive support” OR “cognitive intervention” OR “cognitive exercise” OR “cognitive strategy” OR “cognitive aid” OR “memory function” OR “memory rehabilitation” OR “memory therapy” OR “memory aid” OR “memory group” OR “memory training” OR “memory retraining” OR “memory support” OR “memory stimulation” OR “memory strategy” OR “memory management” OR “brain training” OR “brain rehabilitation” OR “brain stimulation” OR “brain retraining” OR “brain exercise” OR “neuropsychological training” OR “neuropsychological therapy” OR “neuropsychological strategy” OR “neuropsychological aid” OR “neuropsychological stimulation” OR “neuropsychological rehabilitation” OR “neuropsychological exercise” OR “neuropsychological intervention” OR “neuropsychological retraining” OR “neuropsychological support” OR “psychostimulation” OR “executive training” OR “executive stimulation” OR “executive rehabilitation” OR “attention training” OR “attentional training” OR “attentional rehabilitation” OR “global stimulation” OR “reality orientation”
Study terms	RCT OR “controlled trial” OR random*
Subject terms	dement* OR “Alzheimer's disease” OR alz* OR AD OR DAT OR DLB OR FTD OR VD OR “memory impairment” OR “cognitive impairment” OR “memory disorder” OR “cognitive disorder” OR “memory dysfunction” OR “cognitive dysfunction”

AD, Alzheimer's disease; RCT, randomised controlled trial.

### Inclusion and exclusion criteria

Studies were included in the meta-analysis if the study was a peer-reviewed RCT; participants had a diagnosis of dementia; mean age of participants in the study was greater than 60 years; sufficient data were available for calculation of effect sizes (unavailable information was requested from authors and included if obtained); the number of participants in each condition was more than 5 at any point, and standardised general cognitive outcome measures were used. RCTs were included if they compared a cognitive intervention to an active or non-active control or with another treatment (pharmacotherapy or other non-pharmacological therapy). Cognitive interventions were classified as CT, CS or CR^1^ as described in the introduction and in table 1. Studies were independently screened and selected for inclusion and rated as to the best description of the intervention and control groups by three of the authors (JDH, KL, MS), using the criteria described in table 1. If it was decided that a study contained elements of more than one type of intervention it was classed as mixed for example, mixed CT and stimulation (MCTS). Control interventions were classed as either ‘active’ or ‘non-active’ as described in table 1. Disagreements were resolved through discussion with the fourth and fifth authors (RLG and RJH).

### Assessment of trial quality

A risk of bias tool^10^ was used to assess study quality in five areas known to affect clinical outcomes (sequence generation, allocation concealment, blinding of outcome assessors, incomplete outcome data and selective outcome reporting). Studies were independently and blindly rated as to the degree of bias in each study by three of the authors (JDH, KL and MS) and disagreements resolved through discussion with a fourth author (RLG). If a study received an inadequate or unclear rating in all five areas of bias it was excluded from meta-analyses.

### Data extraction

Means and SDs or SEs for each general cognitive outcome measure in each condition and time point were independently extracted for each study by three of the authors (JDH, KL, MS). Disagreements were resolved through discussion with a fourth author (RLG). If means and SD were not available in published articles, authors were contacted and obtained information was included.

### Calculation of effect sizes

Effect sizes (Hedges’ g) were calculated by computing the mean change scores (M_post_−M_pre_ or M_follow-up_−M_pre_) between the intervention and comparator conditions (control or other treatment groups), which allows an estimate of effectiveness even when the intervention and control groups are non-equivalent. The mean change scores were divided by the pooled estimates of the intervention and comparator SDs at preintervention (SDpre) and corrected for positive bias (Cp) to account for bias resulting from small sample sizes. Further details of effect size calculations are found in online supplementary appendix 1.

### Statistical analysis

#### Meta-analyses

Separate meta-analyses were conducted for subtype of cognitive intervention (CT, CS, CR and MCTS), in combination with subtype of control group (active or non-active) and general cognitive outcome measure (MMSE, ADAS-Cog or other), to provide specific pooled effect sizes for each type of intervention and outcome. Separate meta-analyses were also conducted for each outcome measure at different time points (postintervention (defined as 0–4 weeks after the intervention), 3, 6 and 9–12-month follow-up) to avoid non-independence of effect sizes.

### Meta-regression analyses

Planned meta-regression analyses were used to examine whether any between-study heterogeneity could be explained by format of intervention (group or individual) and measures of study quality (sequence generation, allocation concealment, blinding of outcome assessors), as these have been suggested by previous analyses to influence effect size.^7^ Other variables examined were setting of intervention (outpatient/community vs inpatient/care home facilities), intensity of intervention (hours per week), length of intervention (weeks) and severity of dementia (as determined by mean MMSE score).

### Assessment of clinical significance

Mean change scores for each study were also calculated as (M_postintervention_−M_preintervention_)−(M_postcomparator_ −M_precomparator_) to provide estimates that could be directly compared with MCID in outcome measures. The MCID for interventions in dementia has been systematically reviewed elsewhere.^11^ For the ADAS-Cog, the most commonly cited measure, there is general agreement that a 4 point change is clinically significant. There is a greater range of opinion for the MMSE, with values of between 1.4^12^ ^13^ and 3 being cited.^14^ ^15^ Further details of all statistical analyses are found in online supplementary appendix 1.

## Results

### Identification and characteristics of included studies

**Figure 1:**
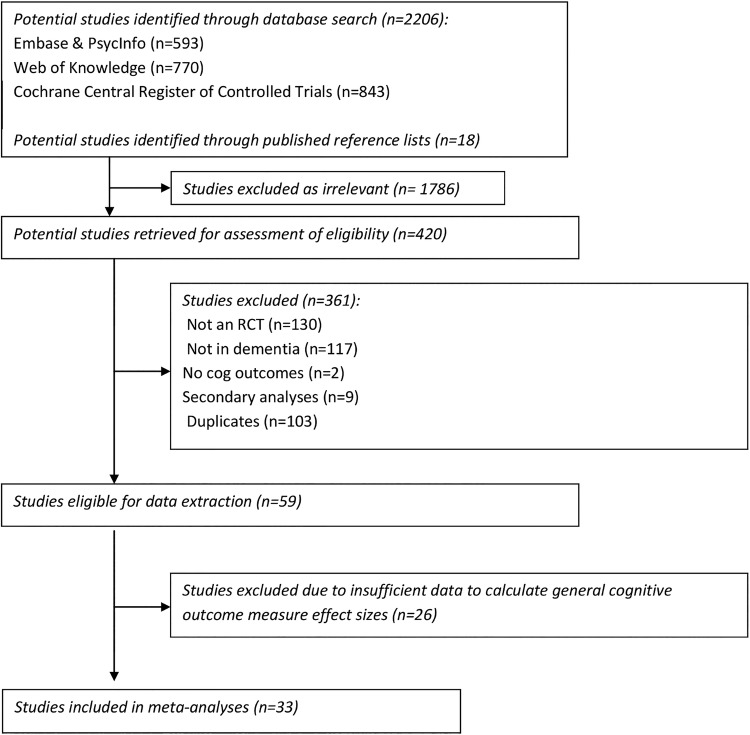
Flow diagram of selection of studies.

Literature searches identified 2206 potential studies, 59 of which met inclusion criteria for data extraction (PRISMA flow diagram, figure 1 and PRISMA checklist, online supplementary table S1). Of these, 33 contained general cognition outcome measures that could be included in meta-analyses and were selected.^16–48^ Summary characteristics of these studies are presented in online supplementary table S2.

Four studies were classified as CT,^16–19^ 21 as CS,^20–40^ and 7 as MCTS.^41–47^ One study contained separate CS and MCTS interventions.^48^ There were no RCTs of CR with general cognitive outcomes.

Only eight studies used active control groups.^16–18^ ^24^ ^25^ ^41^ ^42^ ^46^ Twenty-one studies used non-active control groups, while two studies had active and non-active control groups.^39^ ^47^ One study compared the intervention to other treatments and non-active controls,^33^ and one study compared the same intervention in different settings.^40^

The most commonly used general cognitive outcome measure was the MMSE. Seventeen studies used the MMSE alone,^17–21^ ^23–25^ ^29^ ^36^ ^37^ ^39^ ^42–46^ 10 studies included the MMSE and ADAS-Cog^22^ ^26^ ^27^ ^31^ ^33–35^ ^40^ ^41^ ^48^ and 2 studies used the ADAS-Cog alone as a general cognition outcome measure.^28^ ^30^ Two studies used only other general cognitive measures (Clifton Assessment Schedule (CAS)^38^ and The Mattis Dementia Rating Scale (MATTIS)^47^). Two studies used the MMSE and one other general cognitive measure Milan Overall Dementia Assessment (MODA).^16^ ^32^

Only eight studies included follow-up data,^16^ ^20^ ^26^ ^36^ ^39^ ^47^ ^30^ ^44^ ranging from 6 weeks to 10 months postintervention, with the most common follow-up period being 6 months.

### Quality of studies

Risk of bias and study quality is summarised in online supplementary table S3. Randomisation was the least adequately addressed, with only 12 studies adequately or partially adequately reporting randomisation sequence and 10 studies adequately reporting allocation concealment.

### Meta-analyses of CS studies

**Figure 2:**
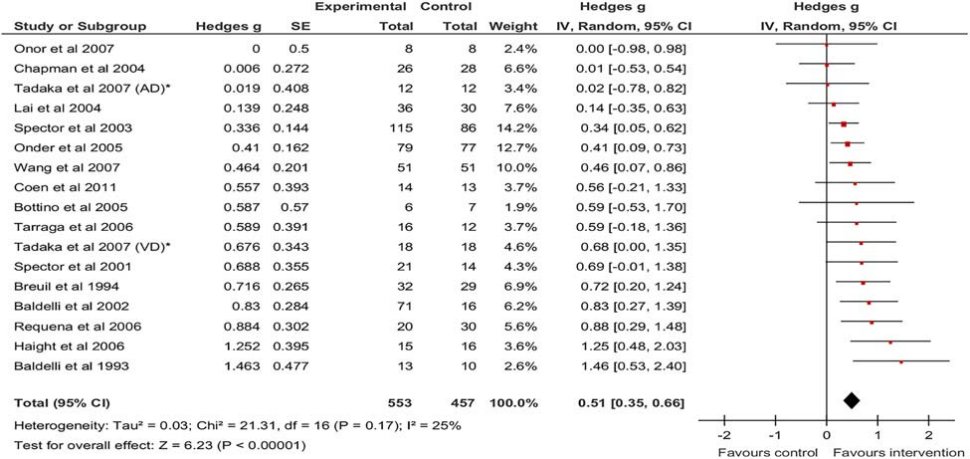
Forest Plot of CS versus non-active controls-MMSE outcome. CS, cognitive stimulation; MMSE, mini-mental state examination.

The results of all meta-analyses conducted are presented in table 3. Postintervention, there was a significant pooled effect size for CS versus non-active controls on the MMSE (g=0.51, 95% CI 0.35 to 0.66, z=6.23, p<0.001, figure 2). There was low heterogeneity between studies (I^2^=24.9%). The calculated 95% prediction interval (0.124 to 0.89) suggested that the intervention was beneficial in individual settings.

**Table 3:** Meta-analyses

Analysis	Number of studies	N in Tx/ control condition	Pooled effect size g (95% CI)*^,^†	Overall effect: Z (p value)	Heterogeneity: *I^2^*% (p Value)	Prediction interval: 95% CI	Publication bias-egger's asymmetry test bias coefficient (p)
*CS*							
Postintervention-MMSE*							
CS vs NA	17	553/457	0.51 (0.35 to 0.66)	6.23 (≤0.001)	24.9 (0.167)	0.12 to 0.89	1.09 (0.14)
CS vs Active	3	108/83	0.35 (0.06 to 0.64)	2.34 (0.019)	0.0 (0.72)	n/a	−2.67 (0.55)
Post-Intervention-ADAS-Cog†							
CS vs NA *No studies of CS* vs *Active*	9	347/313	−0.26 (−0.44 to −0.08)	2.82 (0.005)	18.5 (0.28)	−0.62 to 0.10	−0.017 (0.99)
Postintervention-Other general cog outcome							
CS vs NA	2	21/14	0.25 (−0.44 to 0.94)	0.71 (0.48)	0.0 (0.75)	n/a	UC
Follow-up							
CS vs NA at 3 months (MMSE)	2	49/40	0.80 (0.05 to 1.54)	2.10 (0.036)	54.5 (0.14)	n/a	UC
CS vs NA at 6 months (MMSE)	3	56/58	0.27 (−0.10 to 0.64)	1.45 (0.15)	0.0% (0.61)	n/a	−1.30 (0.76)
CS vs NA at 10 months (ADAS-Cog)	2	76/74	−0.40 (−0.72 to −0.08)	2.41 (0.016)	0.0% (0.38)	n/a	UC
*CT*							
Postintervention-MMSE							
CT vs NA	1	16/16	n/a	n/a	n/a	n/a	UC
CT vs Active *No studies of CT* vs *Active or Non-active using ADAS-Cog*	3	45/42	0.22 (0.75 to 1.18)	0.44 (0.66)	76.9 (0.01)	−11.03 to 11.47	−4.16 (0.61)
*No follow-up studies of CT*							
*MCTS*							
Postintervention-MMSE							
CTCS vs NA	3	41/27	0.45 (−0.57 to 1.46)	0.86 (0.39)	73.8 (0.02)	−11.33 to 12.23	−4.66 (0.79)
CTCS vs active	3	43/41	0.25 (−0.18 to 0.69)	1.15 (0.25)	0.0 (0.39)	n/a	3.74 (0.22)

*For the MMSE outcome, a positive value of g denotes a benefit of the intervention compared with the control.

†For the ADAS-Cog outcome, a negative value of g denotes a benefit of the intervention compared with the control.

ADAS-Cog, Alzheimer's disease Assessment Scale-cognitive subscale; CS, cognitive stimulation; CT, cognitive training; MCTS, mixed cognitive training and cognitive stimulation interventions; MMSE, mini-mental state examination; n/a, not applicable; NA, non-active control group; UC, unable to calculate.

**Figure 3:**
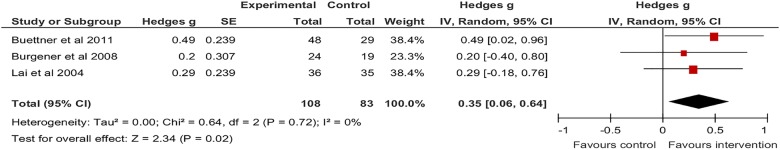
Forest Plot of CS versus active controls-MMSE outcome. CS, cognitive stimulation; MMSE, mini-mental state examination.

A smaller but still significant pooled effect size of 0.35 (95% CI 0.06 to 0.64; z=2.34, p=0.019) was found for CS versus active controls on the MMSE (figure 3), with no heterogeneity between the three studies (I^2^=0.0%).

**Figure 4:**
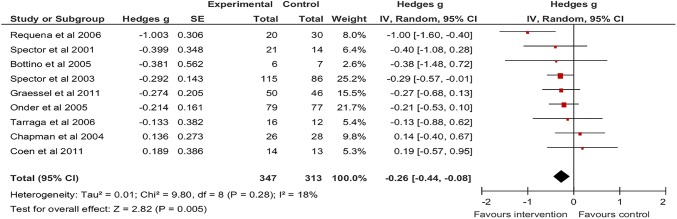
Forest Plot of CS versus non-active controls-ADAS-Cog outcome. ADAS-Cog, Alzheimer's disease Assessment Scale-cognitive subscale; CS, cognitive stimulation.

On the ADAS-Cog there was a significant pooled effect size favouring CS of −0.26 (95% CI −0.44 to −0.08; z=2.82, p=0.005, figure 4). There was low heterogeneity between the nine studies (I^2^=18.5), however, 95% prediction intervals (−0.62 to 0.10) suggested that the intervention may not be beneficial in individual settings. There were no studies comparing CS to active controls that used the ADAS-Cog as an outcome measure.

Two CS versus non-active control studies assessing other general cognitive outcome measures (CAS and MODA) were included in a meta-analysis. A non-significant positive effect size of 0.25 was found (95% CI −0.44 to 0.94; z=0.71, p=0.48).

At up to 3 months follow-up, there was a pooled effect size of 0.796 in favour of CS (95% CI 0.052 to 1.539; z=2.10, p=0.036), on the MMSE, however, both studies included in the analysis compared CS to non-active controls. There was moderate heterogeneity between these studies (I^2^=54.5%). By 6 months follow-up a non-significant pooled effect size of 0.273 (95% CI −0.10 to 0.64; z=1.45, p=0.15) was found on the MMSE, with no heterogeneity between the three studies (I^2^=0.0%). At 10 months follow-up, a significant effect of CS (g=−0.40; 95% CI −0.723 to −0.075; z=2.41, p=0.016) on the ADAS-Cog was seen.

### Meta-analyses of CT studies

Only one study compared CT to a non-active control group,^19^ therefore no meta-analyses could be conducted. On the MMSE there was a non-significant pooled effect size of 0.22 favouring CT versus active controls (95% CI −0.745 to 1.180; z=0.44, p=0.658). There was significant heterogeneity between the three studies (I^2^=6.9%) and 95% prediction intervals (−11.033 to 11.467) suggested that the intervention may not be beneficial in all individual study settings.

There were no studies comparing CT to active or non-active control groups using the ADAS-Cog as an outcome measure. One study used the MODA as an outcome measure therefore no meta-analyses could be conducted.

### Meta-analyses of mixed CT and CS studies

Non-significant pooled effect sizes were found with MCTS versus non-active, g=0.447 (95% CI −0.568 to 1.462; z=0.86, p=0.388) and active controls, g=0.253 (95% CI −0.179 to 0.686; z=1.15, p=0.251) on the MMSE. Heterogeneity between the three MCTS versus non-active control studies was significant (I^2^=73.8%) with 95% prediction intervals (−11.333 to 12.227) suggesting the intervention may not be beneficial in individual settings.

It was not possible to conduct meta-analyses on studies comparing cognitive interventions to other treatments (eg, pharmacological treatment) as only a single study investigated this.^33^ Similarly only a single study compared a cognitive intervention in different settings and therefore no meta-analysis was performed.

### Meta-regression analyses

The results of the meta-regression analyses are presented in table 4.

**Table 4:** Meta-regression analyses

Variables	Regression coefficient (SE)	95% CI	p Value	Q (P)	I^2^
*MMSE outcome studies (n=30)*					
Continuous variables					
Length of intervention (weeks)	0.004 (0.003)	−0.002 to 0.010	0.244	38.3 (0.092)	0.270
Intensity of intervention (h/week)	0.020 (0.019)	−0.018 to 0.059	0.287	38.2 (0.095)	0.267
Severity of dementia (mean MMSE)	0.010 (0.017)	−0.024 to 0.044	0.557	37.9 (0.062)	0.314
Dichotomous variables					
Intervention type (0=CS, 1=CT and MCTS)	−0.163 (0.152)	−0.461 to 0.135	0.284	38.7 (0.086)	0.276
Control type (0=non-active, 1=active)	−0.163 (0.157)	−0.484 to 0.157	0.306	38.6 (0.088)	0.275
Setting (0=outpatient/community, 1=inpatient/care home)	0.153 (0.161)	−0.177 to 0.484	0.349	37.6 (0.065)	0.309
Format (0=group, 1=individual)	−0.233 (0.143)	−0.528 to 0.062	0.116	30.1 (0.147)	0.236
Quality-related dichotomous variables (0=inadequate/unclear, 1=adequate/partially adequate)					
Sequence generation	−0.070 (0.140)	−0.357 to 0.217	0.622	39.3 (0.075)	0.288
Allocation concealment	−0.155 (0.130)	−0.421 to 0.111	0.242	37.9 (0.101)	0.261
Blinding of outcome assessors	−0.184 (0.145)	−0.482 to 0.114	0.216	37.6 (0.105)	0.256
Incomplete outcome data	0.049 (0.150)	−0.257 to 0.356	0.745	39.6 (0.072)	0.293
Selective outcome reporting	−0.289 (0.325)	−0.954 to 0.376	0.381	38.6 (0.087)	0.275
*ADAS-Cog outcome studies (n=11)*					
Continuous variables					
Length of intervention (weeks)	−0.007 (0.003)	−0.014 to 0.0001	0.053	5.19 (0.818)	≤0.001
Intensity of intervention (hours/week)	−0.007 (0.017)	−0.045 to 0.031	0.686	9.93 (0.356)	0.094
Severity of dementia (mean MMSE)	0.004 (0.025)	−0.052 to 0.061	0.860	8.63 (0.374)	0.073
Dichotomous variables					
Intervention type (0=CS, 1=CT and MCTS)	−0.075 (0.381)	−0.937 to 0.788	0.849	10.08 (0.344)	0.107
Control type (0=non-active, 1=active)	0.141 (0.554)	−1.112 to 1.394	0.805	10.05 (0.346)	0.105
Setting (0=outpatient/community, 1=inpatient/care home)	0.184 (0.311)	−0.552 to 0.920	0.573	9.67 (0.208)	0.276
Format (0=group, 1=individual)	0.061 (0.307)	−0.690 to 0.811	0.849	9.60 (0.143)	0.375
Quality- related dichotomous variables (0=inadequate/unclear, 1=adequate/partially adequate)					
Sequence generation	0.239 (0.213)	−0.243 to 0.721	0.291	8.87 (0.450)	≤0.001
Allocation concealment	0.239 (0.213)	−0.243 to 0.721	0.291	8.87 (0.450)	≤0.001
Blinding of outcome assessors	0.408 (0.208)	−0.063 to 0.880	0.082	6.29 (0.711)	≤0.001
Incomplete outcome data	0.270 (0.184)	−0.147 to 0.687	0.177	7.98 (0.536)	0.009
Selective outcome reporting	UC	UC	UC	UC	UC

Q=fit of model without heterogeneity; I^2^=proportion of variation due to heterogeneity.

ADAS-Cog, Alzheimer's disease Assessment Scale-cognitive subscale; CS, cognitive stimulation; MCTS, mixed cognitive training and cognitive stimulation interventions; MMSE, mini-mental state examination; UC, unable to calculate.

For MMSE and ADAS-Cog outcome measures**,** meta-regression analyses revealed no significant associations between effect sizes and type of control group (active vs non-active), setting (inpatient vs outpatient), length of intervention, format of intervention (group vs individual), intensity of intervention in hours per week, or mean severity of dementia of participants. In addition, there were no significant associations between effect sizes and measures of potential bias: randomisation sequence, randomisation allocation, blinding of outcome assessors and incompleteness of outcome data or selective outcome reporting.

The limited number of studies precluded meta-regression analysis at any of the follow-up time points.

### Clinical significance and sensitivity analyses

Repeated random-effects meta-analyses of the main CS comparisons, using SDs of mean change scores, produced a significant weighted mean difference score of 1.78 (95% CI 1.23 to 2.33, p<0.001) for CS versus non-active controls on the MMSE and −1.92 (95% CI −3.43 to −0.4, p=0.01) for CS versus non-active controls on the ADAS-Cog. However, the weighted mean difference score for the MMSE in CS versus active controls was non-significant (1.45, 95% CI −0.11 to 3.02, p=0.07).

Comparisons of the calculated mean change scores for each study with the range of published MCIDs are presented in table 5. For the CS studies, there was only evidence of the majority of studies (11/17) reaching minimal clinical significance with the lowest published threshold for MCID (1.4 MMSE points). However, with the more conservative MCID of >2 MMSE points, only 9/17 CS versus non-active studies and no CS versus active control studies reached MCID. Of note, only 2/9 CS versus non-active control studies reached MCID on the ADAS-Cog.

**Table 5:** Number of studies meeting criteria for MCID

Intervention	Control	MMSE			ADAS-Cog
		MD >1.4*	MD >2†	MD >3‡	MD >4§
CS	NA	11/17	9/17	5/17	2/9
	ACTIVE	2/3	0/3	0/3	0/0
CT	NA	1/1	0/1	0/1	0/0
	ACTIVE	1/3	1/3	0/3	0/0
MCTS	NA	1/3	0/3	0/3	0/1
	ACTIVE	1/3	0/3	0/3	0/1

*Total number of studies reaching MCID of ≥1.4 MMSE points.

†Total number of studies reaching MCID of ≥2 MMSE points.

‡Total number of studies reaching MCID of ≥3 MMSE points. Of note only 5/17 CS versus NA studies and no CS versus active, CT or MCTS studies would reach a MCID of ≥3 MMSE.

§Total number of studies reaching MCID of ≥4 ADAS-Cog points.

ACTIVE, active control group; ADAS-Cog, Alzheimer's disease Assessment Scale-cognitive subscale; CS, cognitive stimulation; CT, cognitive training; MCTS, mixed cognitive stimulation and training; MCID, minimal clinically important difference; MD, mean difference calculated as (M_postintervention_−M_preintervention_)−(M_postcomparator_−M_precomparator_); MMSE, mini-mental state examination; NA, non-active control.

## Discussion

There was evidence of statistically significant efficacy of CS when MMSE was used as the outcome measure, although effect sizes were small to moderate in magnitude (g=0.35 for active and 0.51 for non-active controls). ADAS-Cog score also showed significant improvement in comparisons with only non-active controls, again with a small effect size of g=−0.26. Where there was heterogeneity between the trials reviewed, prediction intervals indicated that CS was beneficial in individual settings as measured by the MMSE but not the ADAS-Cog, which questions the efficacy of CS on ADAS-Cog scores in individual study settings. Our meta-analysis is consistent with recent Cochrane reviews in finding little or no evidence for significant efficacy of CT in dementia, but we further conclude that interventions using a mixture of CT and CS approaches do not significantly improve general cognition.

It is encouraging that for CS there was a statistically significant pooled effect size on general cognitive outcome measures. However, as the effect sizes from CS studies were only small to moderate in magnitude, can we be confident that statistically significant improvements on the outcome measures translate to clinically meaningful benefits in general cognition? Examination of between-group mean MMSE difference scores reveals that only when the lowest threshold for the MCID are used do the majority of studies (11/17 studies) reach minimal clinical improvement. The weighted mean difference for CS studies compared with adequate active controls of 1.45 (95% CI −0.11 to 3.02, p=0.07) only just reaches the lowest MCID threshold of 1.4 points, and was not statistically significant in the sensitivity analysis. When the ADAS-Cog was used as an outcome measure, only two of nine studies versus non-active controls (and no studies vs active controls) demonstrated mean differences of greater than four points, and the weighted mean difference for all studies of −1.92 (−3.43 to −0.4) lies well below the MCID of 4. As there was limited evidence of clinically important differences in MMSE or ADAS-Cog scores when CS was compared to an adequate placebo control, we would conclude that, although statistically significant improvements in MMSE or ADAS-Cog scores are seen with CS, our analysis is consistent with that of Kurz *et al*. We would similarly argue that there is only limited evidence that any cognitive interventions leads to clinically significant cognitive improvement in dementia. The definition of what constitutes a clinically significant difference, however, is open to debate. The MCID values quoted come from pharmacological intervention studies and are limited to the general cognitive outcome measures examined in this meta-analysis. Outcomes not examined in the current study, such as quality of life, functional improvement, mood and carer attitudes are all of definite clinical significance. However, it is valid to compare MCID on general cognitive measures when assessing interventions that aim to improve cognitive function, and therefore taken simply on general cognitive grounds alone, cognitive interventions remain of debatable clinical significance. Further exploration of the clinical significance of improving quality of life in dementia is needed, as well as further qualitative and quantitative analyses examining the effects of interventions to improve quality of life.^49^

A significant issue is the inadequacy of blinding and placebo controls in psychosocial RCTs such as those of CS. The gold standard for pharmacological interventions is the double-blind, active placebo-controlled RCT, with medications not adopted by NICE unless there is sufficient evidence from these types of RCTs. However, the same standards are not held for psychosocial interventions such as cognitive behavioural therapy (CBT) and CS. Consequently, psychosocial interventions may appear more effective than they truly are due to the overestimation of effect sizes resulting from inadequate blinding and placebo controls. Our results suggest this may be a factor, as larger effect sizes were found when CS was compared with non-active controls than when compared with active controls. The meta-regression found that type of control group was not significantly associated with ES; however, this may be due in part to the very small number of studies that used active control groups. Recent meta-analyses of psychological interventions in the elderly have demonstrated that significant effects are not present with active control groups compared with non-active controls.^9^ ^50^ ^51^

Appropriate control interventions need to be designed with care. If the intervention and control groups are too similar to each other, the study may be underpowered to detect an effect. Alternatively, if a control intervention is more enjoyable than the intervention, any true benefits of the intervention may be masked.^52^ However, the alternative of using only non-active or TAU control groups is more problematic, as it could lead to a difference being attributed to the intervention when it may have been due to the non-specific factors of contact, time, socialisation or motivation associated with the intervention.

It is imperative that non-pharmacological interventions are subjected to the same rigour that pharmacotherapy interventions are before recommendations about efficacious treatments can be made, particularly in the current economic climate. At the very least studies examining the true efficacy of psychosocial interventions should aim to be single-blinded (ie, blinding of participants and outcome assessors, but not therapists), active placebo-controlled RCTs. It is, of course, difficult to blind participants in psychosocial interventions, and such blinding raises ethical issues, but it is not impossible, as demonstrated in a recent CBT trial.^53^ Although cognitive measures are performance-based, the knowledge that a participant is engaged in a placebo condition may have effects on engagement or motivation that could impact performance during the intervention and on outcome measures. There would be clear ethical issues in misleading participants into believing that a control is actually an intervention. However as the ‘active ingredients’ of efficacious cognitive interventions become clearer, a move to more comparative studies would be useful, with different training or stimulation programmes compared.

Ultimately, it is clear from our meta-analyses that more randomised, single-blind, active placebo controlled studies are required to properly assess the efficacy of cognitive interventions in dementia.

### Limitations

A difficulty in the literature lies in characterising the content of the interventions. We based our classifications on the definitions suggested by Clare and Woods*l*,^1^ however, a wide range of differing content remained. Within CS, different approaches included reality orientation, reminiscence therapy, socialisation activities, use of external memory aids and physical exercise. As well as differing settings and formats, which could be quantified, there were also differences that were more difficult to quantify to allow formal analysis such as encouraging techniques to be practiced between sessions, motivation of participants, encouraging carer involvement and educating carers. We decided to include studies that appeared to use predominantly differing methods (eg, reality orientation and reminiscence therapy) within CS. It could be argued that it is not appropriate to directly compare studies with differing methodologies, or include studies that have a very wide range of interventions (eg, those that include physical and social as well as cognitive elements), due to the inability to isolate the active ingredient in these regimes. However, despite the wide variety of content, the studies included in our analyses all aimed to improve cognition by delivering a cognitive intervention and could be characterised according to the criteria stated in table 1. It could also be argued that some studies we have classed as mixed, could be classified as either CT or CS, as other authors have done. However, by creating a category of mixed CS and CT our aim was to reduce heterogeneity between studies, to improve the quality of the meta-analyses.

A similar difficulty is in assessing the quality and nature of control interventions. There are a wide variety of control interventions in the literature and some may be criticised for being poorly structured, inconsistently delivered or not theoretically based. Placebo interventions that are poorly conceived, designed or delivered would clearly be inadequate controls. Therefore there is a need for theoretically based, well designed, blinded and adequate active control interventions, rather than relying on non-active TAU controls, which in themselves may differ widely from each other.

In defining control groups as ‘active’ we acknowledge there may be heterogeneity between the active controls used in different studies. However, in our opinion, basing our classification on definitions in table 1, and analysing active controls separately from non-active controls provided a more precise estimate of the efficacy of cognitive interventions, than when all studies are included in the same meta-analysis irrespective of the type of control group.

A further limitation of our study was only analysing MMSE and ADAS-Cog as outcome measures. This was based on the pragmatic need to examine clinically useful and recognised measures of general cognitive function. It is therefore beyond the scope of this meta-analysis to comment on whether a particular cognitive intervention may have significant benefits that could not be identified using the MMSE or ADAS-Cog. This is likely to apply to CT that may aim to improve function in a specific cognitive domain such as executive function, language or episodic memory. There may be benefits within these domains as a result of training that would be apparent if measured on more specific standardised tasks, but may not lead either to general cognition improvement, or improvements may not be seen on measures such as the MMSE or ADAS-Cog which may not be sufficiently sensitive to change.^54^ However, if these non-pharmacological interventions are to be recommended as ‘cognitive’ therapies for patients with dementia it is important to evaluate the evidence for their efficacy on overall cognition as well as on specific standardised tests.

In conclusion, this meta-analysis and meta-regression has shown that CT or combined MCTS interventions do not improve general cognition in patients with dementia. There is evidence that CS programmes can improve MMSE and ADAS-Cog scores, however, heterogeneity means that CS may not show benefit on the ADAS-Cog in all settings, and improvements on the ADAS-Cog are not generally clinically significant. The limited number of studies that include adequate active control conditions and lack of double blinding also make it difficult to compare efficacy with pharmacological interventions.
